# How It All Began

**DOI:** 10.3390/e22080908

**Published:** 2020-08-18

**Authors:** R. Stephen Berry, Peter Salamon, Bjarne Andresen

**Affiliations:** 1 Department of Chemistry, The University of Chicago, 5735 S. Ellis Ave, Chicago, IL 60637, USA; dont-use@uchicago.edu; 2 Department of Mathematics and Statistics, San Diego State University, 5500 Campanile Drive, San Diego, CA 92182-7720, USA; salamon@sdsu.edu; 3 Niels Bohr Institute, University of Copenhagen, Blegdamsvej 17, DK-2100 Copenhagen Ø, Denmark

The first paper published as *Finite-Time Thermodynamics* is from 1977 [1]. It was preceded by the 1975 article by Curzon and Ahlborn entitled “Efficiency of a Carnot engine at maximum power output” [2]. But of course the climate for such a development had to be ripe. Below we each give our personal recollections about how we got started on this endeavor. This is not intended solely as a historical note, but also as a reminder to younger enthusiastic people that the road for new ideas that disturb the traditional lines of thought often is bumpy, if not outright hostile. But there is light at the end of the tunnel!

Shortly before the deadline, Steve Berry passed away. We have kept his recollections below exactly as he originally wrote them, except for correcting a single dating. We dedicate this special Finite-Time Thermodynamics issue to Prof. R. Stephen Berry for his vision, contributions, and perpetual assistance.

## 1. R. Stephen Berry

The topic—or field—of finite time thermodynamics has an interesting history. Its stimulus was a far cry from a motivation to do basic science. Its real origins began when I moved to The University of Chicago in 1964. I had thought I was prepared to adapt to the Chicago environment, but it turned out otherwise. At that time, Chicago was a very smoky, dirty, even smelly city. Each morning, windowsills had new layers of fine grit that had drifted in from the outside during the night. I found myself angry that my new city could have such terrible air pollution. I was sufficiently troubled that I wrote a letter to then-Mayor Richard Daley, which began, “Dear Mayor Daley, You live like a pig!” I went on to say that I had heard that the City of Chicago did have some activity to address air pollution, but I could see no sign of it. I received a reply with an invitation to visit the City’s Air Pollution Laboratory. The visit left me with a sense that nothing substantive was happening to address the problem. I wrote an article [3], “Perspectives on Polluted Air”, which pointed out the untoward consequences of severe air pollution, such as the human death rate in incidents of high levels of air pollution. I also became involved in some of the public anti-air pollution activities that were stirring at the time, activities that, nationwide, led to a “tipping point” that resulted in a national transformation, with the passing in 1967 of the Federal Air Quality Act and then the Clean Air Act, and the creation of the Environmental Protection Agency.

In that period of transformation, I came to believe that one necessary transformation to improve air quality would be to use energy more efficiently than we had been doing. Much of the pollution came from energy production and much from its end use. I was not alone by any means in my concern with the problem [4]. I was sufficiently concerned that, with my student Peter Lehman, we wrote a review article on the chemistry of air pollution [5]. But the “big step” for me came when Margaret Fels and I did a detailed study to show a way to identify likely targets for improving efficiency of energy use [6,7]. We chose the manufacture and disposal of automobiles as the subject and examined each step, from recovery of ore in the ground to final disposal of a used-up hulk, determining the actual energy and free energy at each step, and comparing those values with theoretical ideal thermodynamic limits. Of course we supposed that where the differences were largest, would be the best opportunities for improving the efficiency. This kind of approach was just beginning and has become what is now called “life cycle analysis”.

We thought this was rather a ground-breaking approach and applied the method to other systems, such as packaging, transporting and marketing consumer goods [8], and to polymers [9]. However, one person who had read the article said to me, “Why did you compare the actual energy and free energy use with the ideal thermodynamic limits? After all, those limits are based on reversible, infinitely slow processes. Who would wait for delivery of a car from a manufacturer who claimed to make his cars reversibly?” That challenge turned out to be the trigger!

In 1975 we began exploring these questions: “Is it possible to create, construct and evaluate the analogues of thermodynamic potentials for processes constrained to operate in finite times?” The first venture, carried out with postdoctoral associates Abraham Nitzan and Bjarne Andresen and graduate student Peter Salamon, made use of a model, a variant of the Carnot cycle in which the system moves around in a series of stepwise small pressure changes, relaxing at a finite rate to each new pressure [2]. Thus the system goes around its cycle in a finite number of steps, relaxing to each new pressure in a small, finite time. Very artificial, yes, but a model that lends itself to modeling a thermodynamic system that evolves in finite time, the relaxation times following each sudden stepwise change in pressure.

Very soon after that initial venture, we moved forward in more substantive ways. Within months of that first paper, we made what I feel was a major step, showing how one could create the analogues of traditional thermodynamic potentials for systems constrained to carry out their cycles in fixed, finite times [10], and then how to determine extremal values for finite-time processes [11]. That led naturally to a general method for optimization [12]. A slight pause and a new flow of publications began. When Mary Ondrechen joined the group, we began applying finite-time thermodynamics to chemical processes [13,14].

Our investigations broadened at that time, first to relate our work to a geometric concept from Frank Weinhold [15] and then to explicit optimization. First minimize entropy production [16] and then to improving performance of piston engines by controlling the piston’s path in time [17]. We soon pursued that direction for both Otto and Diesel cycles but never found a practical way to achieve those optimal time paths.

At that time, finite-time thermodynamics had become a topic of investigation for enough people that we began holding Summer Conferences. The first two were at the Aspen Center for Physics, in 1981 and 1983. That second one was so popular that the participants didn’t want to skip a year. However, the Aspen Physics Center’s schedule was already full so we couldn’t hold a meeting there in 1984. Peter Salamon and I were discussing this problem when he said, ‘We both know Telluride; why don’t we see if we can hold the meeting there?” I thought that was a brilliant idea, so we went ahead with it and, in the summer of 1984, held the first Telluride scientific conference. It was at least as popular as the Aspen meetings had been, and they have continued and stimulated many other kinds of meetings in Telluride. They mostly fall in a general category of “molecular science” so they are complementary, rather than competitive, with the Aspen Physics Center’s meetings.

So we can see how anger at an unpleasant, even dangerous environment can have amazing and totally unexpected consequences.

*“Acknowledgement—I would like to thank all the collaborators who have worked with me in creating, developing and applying finite-time thermodynamics, perhaps most notably Peter Salamon and Bjarne Andresen, with whom I am collaborating to create this volume.”*

## 2. Peter Salamon

As a freshman graduate student looking for an advisor to work with, I was looking for one to underwrite a project exploring the differential geometry of thermodynamics. When I approached Steve Berry, he responded with a question, “While you’re at it, can you put time in?” It was spring of 1973 and I had found my mentor.

The notion seemed intriguing. Having been raised in the deeply structuralist traditions prevailing in mathematics departments in the 1970s, it seemed likely to me that understanding the mathematical structure of thermodynamics would enable us to see how this structure might accommodate time. I had studied the differential geometrical framework of classical mechanics and knew how time dependence changes the symplectic structure on the manifold of configurations into a contact structure. I thought there was a good chance of finding something similar for thermodynamics.

Steve however did not conceive of the “add time” dictum in terms of the mathematical structure it would require. Rather he asked the much more physical question of what the constraint of a given time implied for a thermodynamic process. He also pointed us toward heat engines and drafted Abraham Nitzan who was doing a postdoc across town and Bjarne Andresen who joined in the summer of 1975. We learned of Curzon and Ahlborn’s result from one of the more encouraging early referees, who rejected our work as legitimate new physics but suggested that we should consider it as physics education. What Steve’s account of “How it began” did not mention about this nascent stage in the life of the subject was the initial hostility to the idea of finite-time thermodynamics on the part of referees and by extension the community. I feel this is an important historic detail for early career scientists to take heart from. I know I would likely not have survived the onslaught of rejections without Steve’s staunch support.

Due to our years of abuse at the hands of referees, the subject did not seem fully legitimate to me until I could see the broader responses from the community. For this, the Aspen workshops in 1981 and 1983 were crucial. As I was driving home from the 1983 workshop, I came up with a proof of the horse–carrot theorem [18] and wanted to organize a workshop in 1984 on the many directions the result could lead. Alas, Steve told me Aspen was full. Bjarne was spending his sabbatical with me in San Diego, and with his help we organized the first Telluride workshop.

## 3. Bjarne Andresen

In 1975 I came to Chicago to work with Steve, financed by a grant from the Danish Science Foundation to study atomic collision theory, my previous specialty. Within a week, discussions with Steve and Peter had gotten me hooked on the idea that developing a more realistic form of thermodynamics than the standard reversible theory was a brilliant idea. After all, we had just had the oil crisis of 1973 with oil rationing and car-free Sundays in Europe. That started a year of enthusiastic search for consequences of adding that small detail of a finite time horizon for processes, “the cost of haste”. At the end of the year I had to report to the Foundation that, sorry, I did not do anything of what you paid me for but I started this new project instead. No comment whatsoever, just “Thank you for the report”. I am sure that wouldn’t have worked today, but they did the right thing.

Our first four publications on Finite-Time Thermodynamics [1,10,11,12] carry the year 1977 even though all the work was done during this first year. The problem, as Peter also mentions above, was solid resistance from the established community. Extreme doubt at the first conference we presented the ideas [12] could be interpreted positively as “inspirational”. But I had never imagined the solid opposition we met from the editors of several major journals. Later I have observed that such resistance to modifying established ideas is ubiquitous. The main point of telling youngsters about our trials and tribulations is to help them understand exactly this human trait. What we did simply could not be true because it was not within the standard teachings of thermodynamics. It took two years of fighting referees and editors to get those initial papers out. Some colleagues were so adamant in their efforts to eradicate these new thoughts that they wrote to editors of several journals trying to use their standing to convince the editors to reject anything with the words finite-time thermodynamics in the title outright, content unseen. However, that did not prevent some of those colleagues from taking our general proofs, restricting their coverage, and then publishing them as their own original discoveries, under a new name of course. At another incident in 1981, at a luncheon with a Nobel laureate, he characterized the finite-time results as “either useless or something that I have already published”. It was not until Physics Today in 1984 ran a news article [19] on the finite-time concept that recognition began. So, young scientists with all your bright and unorthodox ideas, beware. You have an uphill battle ahead of you, but don’t give up.

During a subsequent stay in Chicago in 1977, we had started determining the process paths which would yield the optimal performance. As an enthusiastic young man I had invited myself to the General Motors Research Laboratories in Detroit, a short flight from Chicago, to offer them the great news that the ordinary gasoline engine could be made 15% more fuel efficient and at the same time cut the cooling requirement in half simply by moving the piston a little differently in each stroke [17]. I was well received and a group of engine design engineers listened patiently to my presentation. At the end the head of the department thanked me for coming over, but he did not want to get further into such a scheme because, as he said, “people are happily buying our current cars, so why should we change them”? This is just another version of the publication resistance to novel ideas mentioned above.

From the beginning we wanted to investigate the effects of requiring a finite time horizon for any process as widely as possible. Some efforts resulted in completely general principles involving duration dependent chemical potentials [10,20]. Others lead to the definition of a thermodynamic length [15,18] which ties dissipation, and thus the finite time, to a geometric formulation. The new concepts have been applied not just to thermodynamics but also to engineering [21], statistical mechanics [22], optimization theory, [23] chemical reactions [24], quantum mechanics [25], biology [26], and economics [27], just to mention some that we have been involved in ourselves. A major conceptual discovery, not related to the dissipation itself, is that it is possible to accurately measure changes in free energies, i.e., equilibrium properties of substances, through non-equilibrium measurements using, e.g., the Jarzynski equation [28] and the Crooks theorem [29]. A book [30] and two review articles [31,32] have summarized the FTT results so far.

It has been a pleasure to be along for this long and inspiring ride and I thank all the wonderful people I have encountered along the way. Let this be an encouragement to all young people to believe in their grand ideas and explore them in the face of opposition. Future paradigms must all pass through a heresy stage.

## References

[1] Andresen B., Berry R.S., Nitzan A., Salamon P. (1977). Thermodynamics in finite time. I. The step-Carnot cycle. Phys. Rev. A.

[2] Curzon F.L., Ahlborn B. (1975). Efficiency of a Carnot engine at maximum power output. Am. J. Phys..

[3] Berry R.S. (1970). Perspectives on Polluted Air. Bull. At. Sci..

[4] Anastaplo G., Berry R.S., Coase R.H., Demsetz H., Friedman M. (1970). The Legal and Economic Aspects of Pollution.

[5] Berry R.S., Lehman P.A. (1971). Aerochemistry of air pollution. Annu. Rev. Phys. Chem..

[6] Berry R.S., Fels M.F. (1972). The Production and Consumption of Automobiles, and Energy Analysis of the Manufacture, Discard and Reuse of the Automobile and Its Component Materials. Report to the Illinois Institute for Environmental Quality.

[7] Berry R.S., Fels M.F. (1973). The energy cost of automobiles. Bull. At. Sci..

[8] Berry R.S., Makino H. (1974). Energy Thrift in Packaging and Marketing. Technol. Rev..

[9] Berry R.S., Long T.V., Makino H. (1975). Energy Budgets 5. An international comparison of polymers and their alternatives. Energy Policy.

[10] Salamon P., Andresen B., Berry R.S. (1977). Thermodynamics in finite time. II. Potentials for finite-time processes. Phys. Rev. A.

[11] Andresen B., Salamon P., Berry R.S. (1977). Thermodynamics in finite time. Extremals for imperfect heat engines. J. Chem. Phys..

[12] Andresen B., Berry R.S., Salamon P., Fazzolare R., Smith C.B. (1977). Optimization of processes with finite-time thermodynamics. International Conference on Energy Use Management.

[13] Ondrechen M.J., Berry R.S., Andresen B. (1980). Thermodynamic in finite time: A chemically driven engine. J. Chem. Phys..

[14] Ondrechen M.J., Andresen B., Berry R.S. (1980). Thermodynamics in finite time: Processes with temperature-dependent chemical reactions. J. Chem. Phys..

[15] Salamon P., Andresen B., Gait P.D., Berry R.S. (1980). The significance of Weinhold’s length. J. Chem. Phys..

[16] Salamon P., Nitzan A., Andresen B., Berry R.S. (1980). Minimum entropy production and the optimization of heat engines. Phys. Rev. A.

[17] Mozurkewich M., Berry R.S. (1981). Finite time thermodynamics: Engine performance improved by optimized piston motion. Proc. Nat. Acad. Sci. USA.

[18] Salamon P., Berry R.S. (1983). Thermodynamic length and dissipated availability. Phys. Rev. Lett..

[19] Andresen B., Salamon P., Berry R.S. (1984). Thermodynamics in finite time. Phys. Today.

[20] Andresen B., Rubin M.H., Berry R.S. (1983). Availability for finite-time processes. General theory and a model. J. Phys. Chem..

[21] Schaller M., Hoffmann K.H., Rivero R., Andresen B., Salamon P. (2002). The influence of heat transfer irreversibilities on the optimal performance of diabatic distillation columns. J. Non Equilib. Thermod..

[22] Salamon P., Nulton J.D., Berry R.S. (1985). Length in Statistical Thermodynamics. J. Chem. Phys..

[23] Salamon P., Nulton J., Robinson J., Pedersen J., Ruppeiner G., Liao L. (1988). Simulated Annealing with Constant Thermodynamic Speed. Comput. Phys. Commun..

[24] Bak T.A., Salamon P., Andresen B. (2002). Optimal behavior of consecutive chemical reactions A<=>B<=>C. J. Phys. Chem. A.

[25] Insinga A., Andresen B., Salamon P., Kosloff R. (2018). Quantum heat engines: Limit cycles and exceptional points. Phys. Rev. E.

[26] Roach T.N.F., Salamon P., Nulton J., Andresen B., Felts B., Haas A., Calhoun S., Robinett N., Rohwer F. (2018). Application of finite-time and control thermodynamics to biological processes at multiple scales. J. Non-Equil. Thermod..

[27] Tsirlin A., Gagarina L. (2020). Finite-Time Thermodynamics in Economics. Entropy.

[28] Jarzynski C. (1997). Nonequilibrium Equality for Free Energy Differences. Phys. Rev. Lett..

[29] Crooks G.E. (1998). Nonequilibrium Measurements of Free Energy Differences for Microscopically Reversible Markovian Systems. J. Stat. Phys..

[30] Berry R.S., Kazakov V., Sieniutycz S., Szwast Z., Tsirlin A.M. (2000). Thermodynamic Optimization of Finite-Time Processes.

[31] Hoffmann K.H., Burzler J.M., Schubert S. (1997). Endoreversible Thermodynamics. J. Non Equilib. Thermodyn..

[32] Andresen B. (2011). Current Trends in Finite-Time Thermodynamics. Angew. Chem. Int. Ed..

